# Supplementary material for: Exploiting the Stemness and Chemoresistance Transcriptome of Ewing Sarcoma to Identify Candidate Therapeutic Targets and Drug-Repurposing Candidates
Source: Cancers (Basel). 2023 Jan 26;15(3):769. doi: 10.3390/cancers15030769 (PMC9913297; doi:10.3390/cancers15030769)
Supplement: Supplementary file 1 [file cancers-15-00769-s001.zip › cancers-2050809-WB.pdf]

Figure 2A

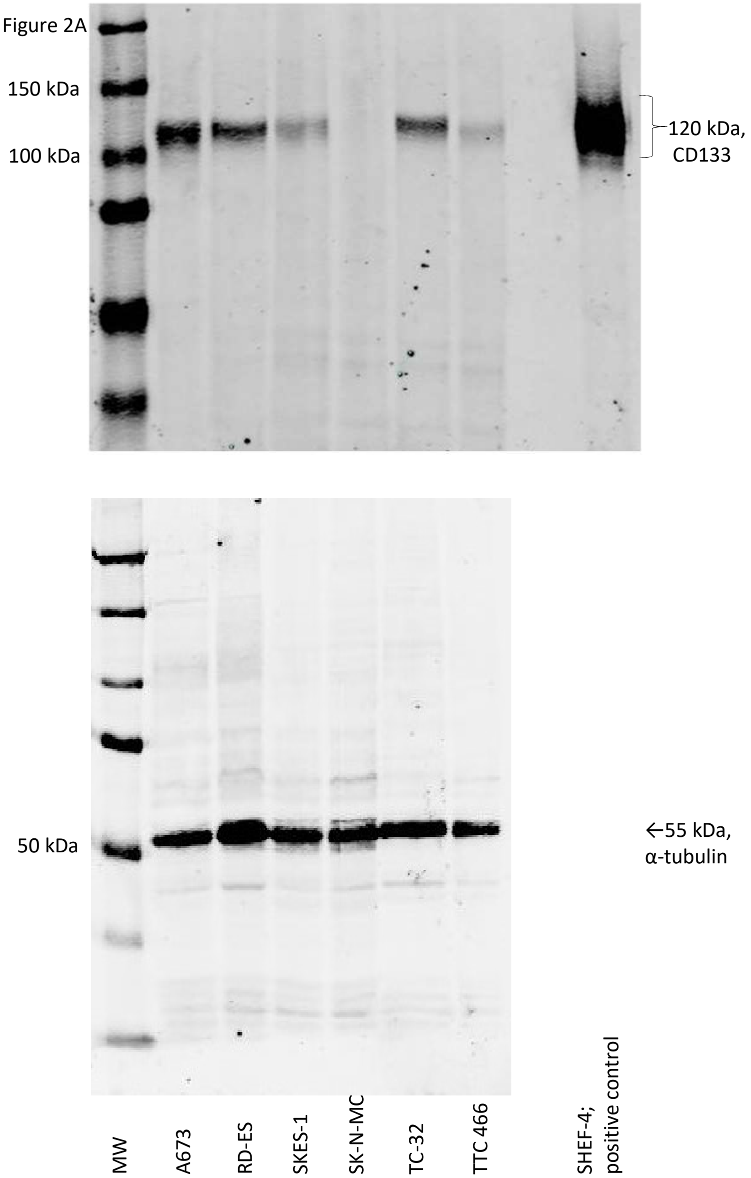

Supplementary Figure 2-A673

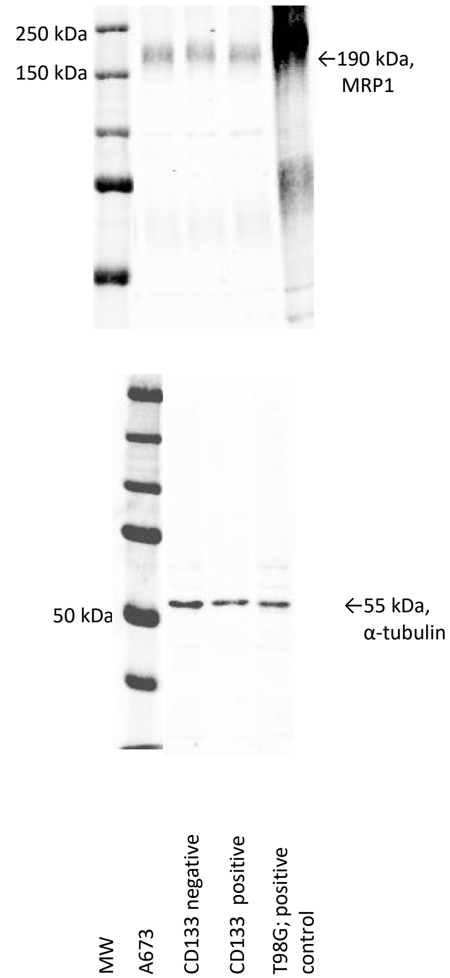

Supplementary Figure 3- TC32

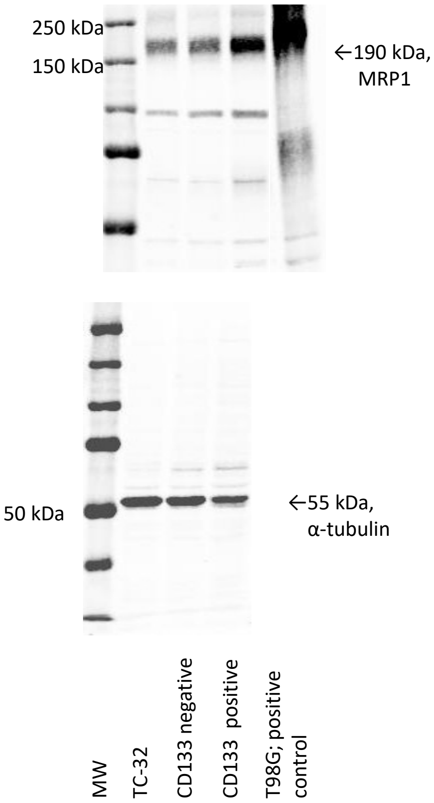

Supp 2G and 3G

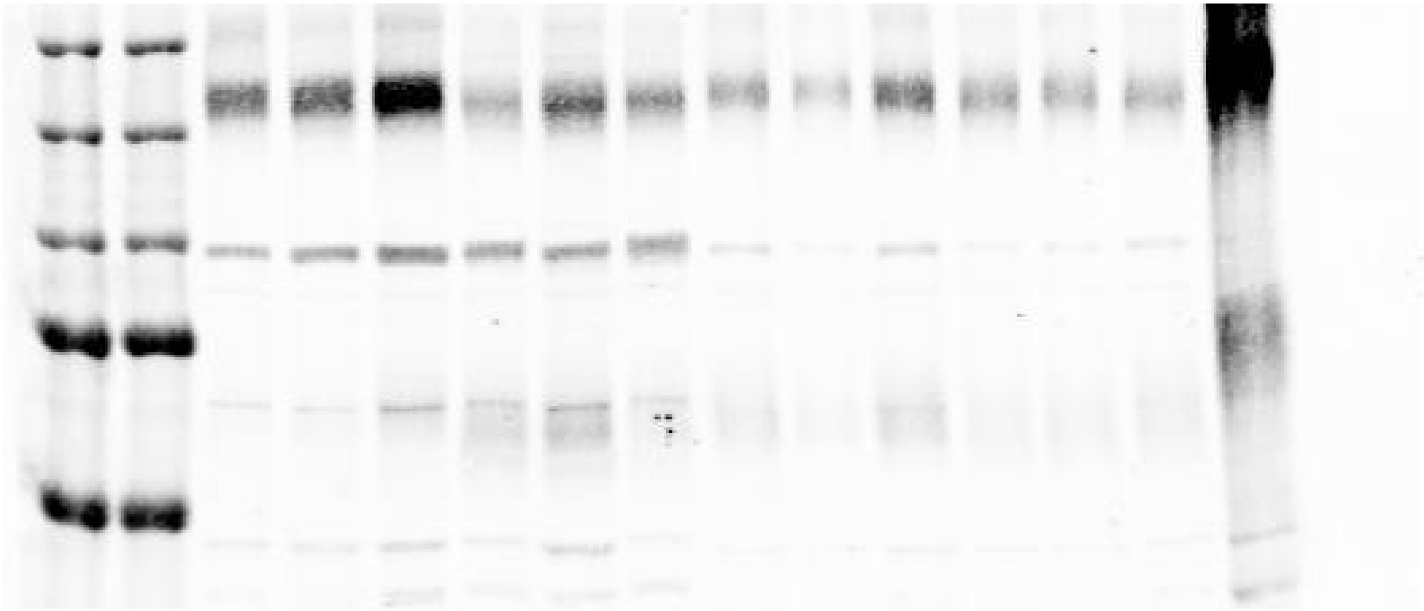

Supp Figure 3G
TC32

Supp Figure 2G
A673

Figure 4I

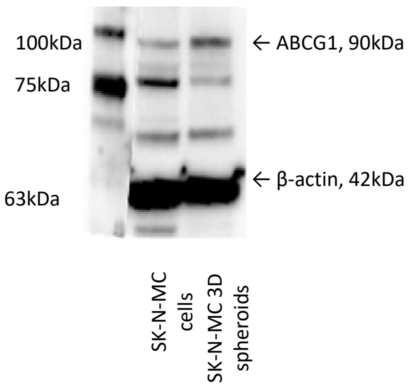
100kDa
75kDa
63kDa
← ABCG1, 90kDa
← β-actin, 42kDa
SK-N-MC cells
SK-N-MC 3D spheroids

Figure 6D

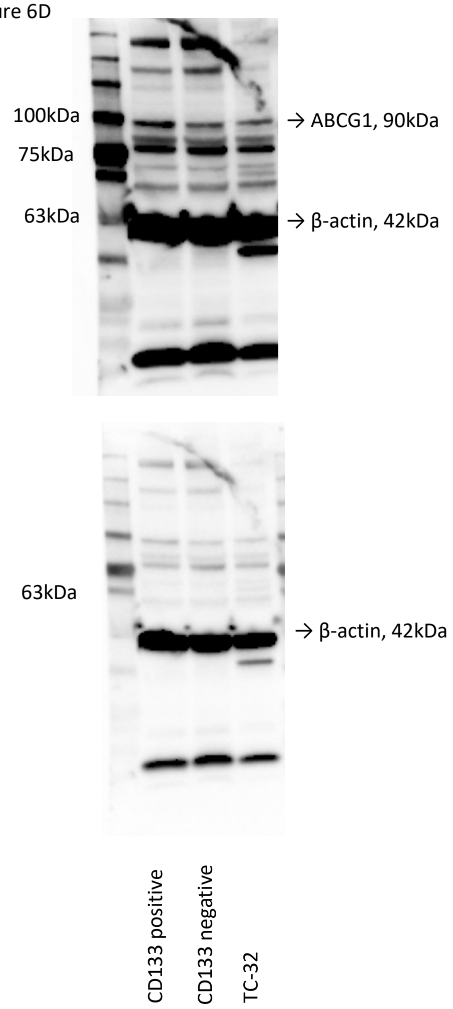

Figure 6E

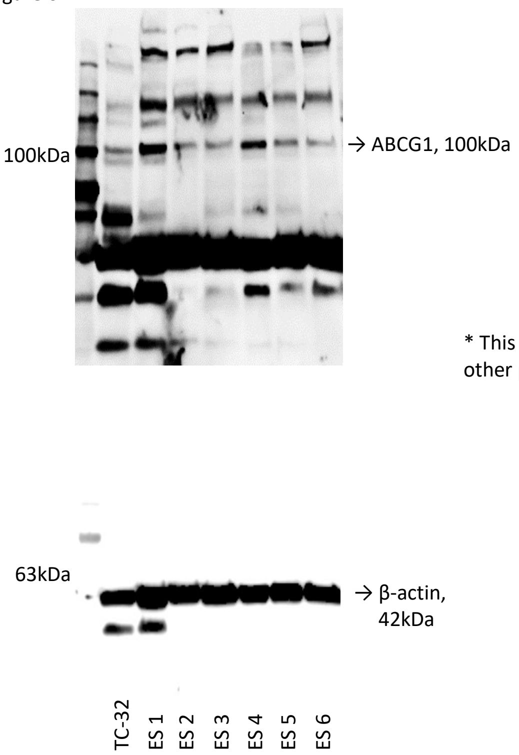

* This WB was probed for other antibodies for other projects
