# Supplementary material for: Exploiting the Stemness and Chemoresistance Transcriptome of Ewing Sarcoma to Identify Candidate Therapeutic Targets and Drug-Repurposing Candidates
Source: Cancers (Basel). 2023 Jan 26;15(3):769. doi: 10.3390/cancers15030769 (PMC9913297; doi:10.3390/cancers15030769)
Supplement: Supplementary file 1 [file cancers-15-00769-s001.zip › Supplementary Data 1.pdf]

Supplementary Data 1

CD133 was expressed on the cell surface of more than 5% of 4/6 ES cell lines (A673, RD-ES, SKES-1, TC-32, mean percentage positive CD133 cells was 12±4, range=1-40%). A673 cells contained the greatest proportion of CD133 expressing cells (40±2% positivity, $p<0.05$).

A673 and TC-32 CD133-positive cells formed significantly more colonies in soft agar (19±4% and 16±1%, respectively) than the CD133-negative cells (5±1% and 3±1%, respectively, $p<0.0001$, Supplementary Data 2 and Supplementary Data 3). There was no difference in proliferation, increased viable cell number, cell cycle status or telomere length in CD133-positive and -negative A673 (Supplementary Data 2) or TC-32 cells (Supplementary Data 3). Interestingly, migration was increased in TC-32 CD133-negative cells compared to that of CD133-positive cells ($p<0.05$; Supplementary Data 3F). There was no significant difference in the migration of CD133-positive compared to CD133-negative A673 cells (Supplementary Data 2F). This was independent of the expression of the polycomb genes BMI-1 and EZH2 (results not shown), previously linked with migration of ES cells and reported to be overexpressed in CSCs [1,2].

Expression of the multi-drug resistant protein MRP1 was increased in CD133-positive TC-32 cells compared to CD133-negative cells (Supplementary Data 3G). Higher expression of MRP1 in TC-32 cells was associated with increased efflux activity ($p<0.01$, Supplementary Data 3H) and resistance to the MRP1 substrate doxorubicin ($p<0.012$, Supplementary Data 3I). However, MRP1 was not increased in the A673 CD133 positive cells (Supplementary Data 2G), resulting in no significant increase in efflux activity (Supplementary Data 2H) or resistance to doxorubicin (Supplementary Data 2I).

References

1. Kang, Y. Analysis of cancer stem cell metastasis in xenograft animal models. *Methods Mol Biol* **2009**, *568*, 7-19, doi:10.1007/978-1-59745-280-9_2.
2. Sampieri, K.; Fodde, R. Cancer stem cells and metastasis. *Semin Cancer Biol* **2012**, *22*, 187-193.
