# Supplementary material for: Exploiting the Stemness and Chemoresistance Transcriptome of Ewing Sarcoma to Identify Candidate Therapeutic Targets and Drug-Repurposing Candidates
Source: Cancers (Basel). 2023 Jan 26;15(3):769. doi: 10.3390/cancers15030769 (PMC9913297; doi:10.3390/cancers15030769)
Supplement: Supplementary file 1 [file cancers-15-00769-s001.zip › Supplementary Data 2_3_4_5_6_9.pptx]

## Slide 1
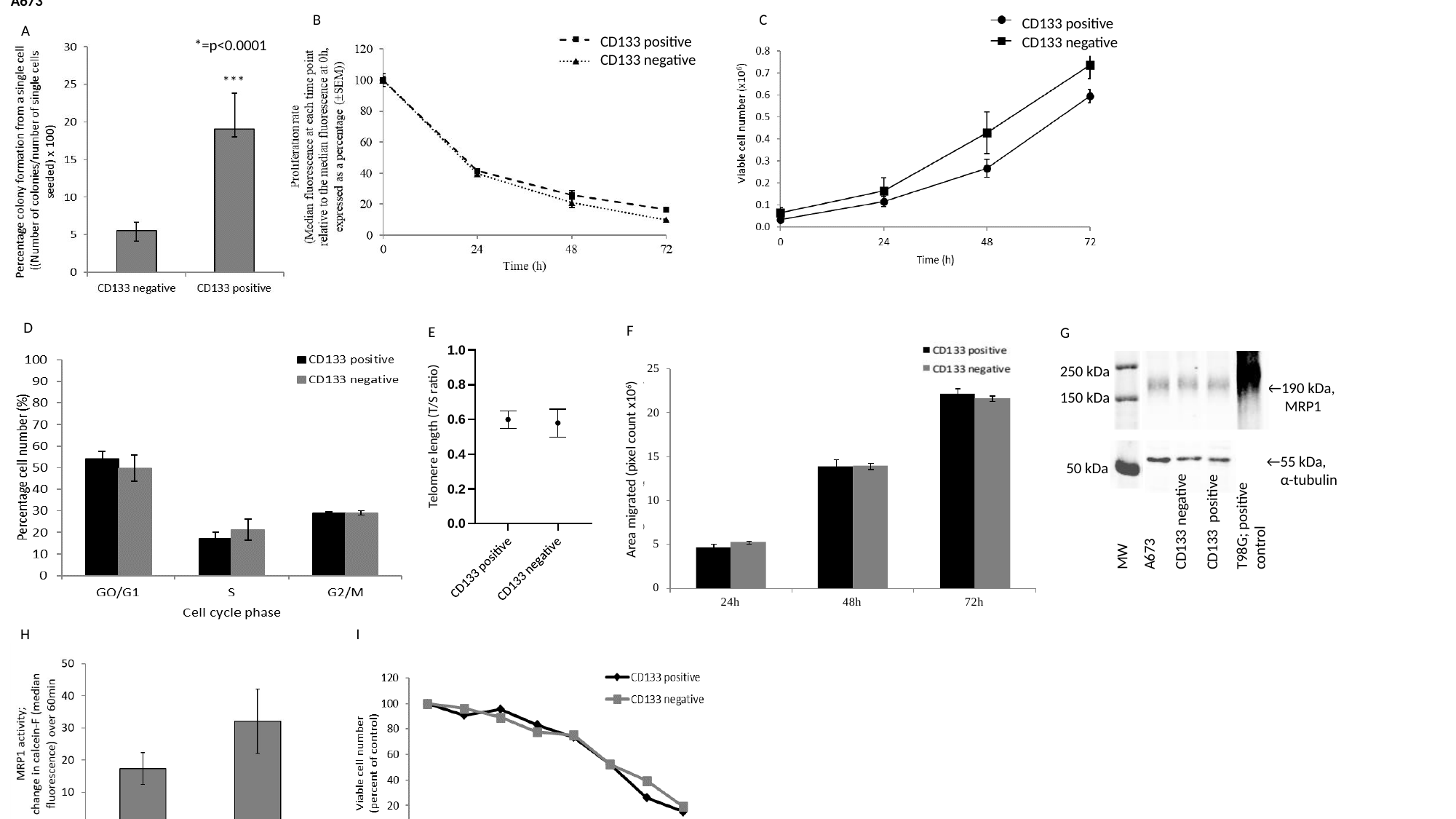

Supplementary Data 2
A673
CD133 positive
CD133 negative
C
B
CD133 positive
CD133 negative
*=p<0.0001
***
A
D
F
Area migrated (pixel count x106)
E
G
250 kDa
←190 kDa,
 MRP1
150 kDa
←55 kDa,
 α-tubulin
50 kDa
T98G; positive control
CD133 positive
A673
CD133 negative
MW
I
H

## Slide 2
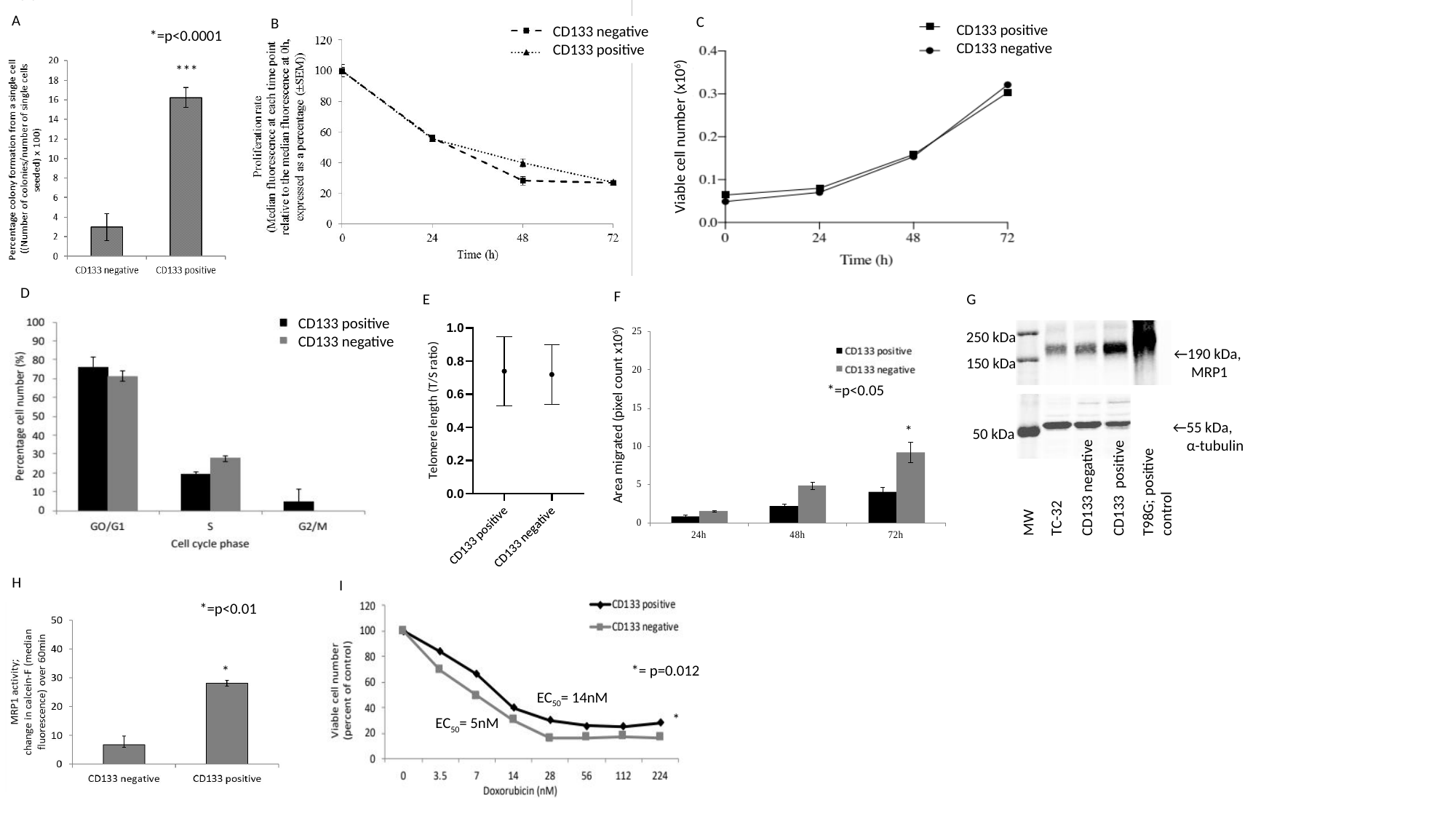

Supplementary Data 3
TC-32
CD133 negative
CD133 positive
B
A
*=p<0.0001
***
C
Viable cell number (x106)
CD133 positive
CD133 negative
D
CD133 positive
CD133 negative
F
*=p<0.05
*
Area migrated (pixel count x106)
G
250 kDa
←190 kDa,
 MRP1
150 kDa
←55 kDa,
 α-tubulin
50 kDa
T98G; positive control
CD133 positive
CD133 negative
TC-32
MW
E
H
*=p<0.01
*
I
*= p=0.012
EC50= 14nM
EC50= 5nM
*

## Slide 3
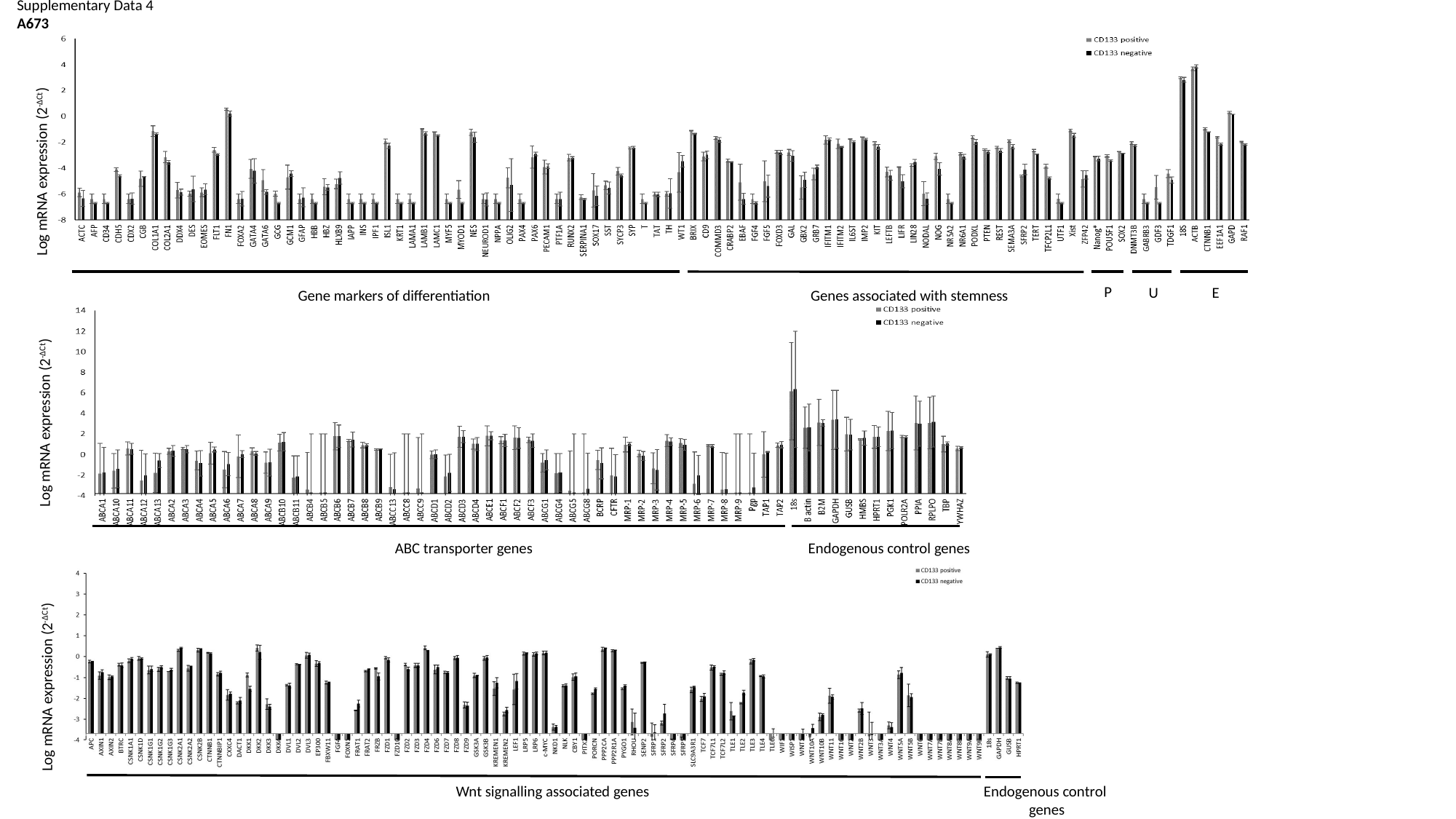

Supplementary Data 4
A673
P
U
E
Genes associated with stemness
Gene markers of differentiation
Log mRNA expression (2-∆Ct)
Log mRNA expression (2-∆Ct)
ABC transporter genes
Endogenous control genes
Log mRNA expression (2-∆Ct)
Wnt signalling associated genes
Endogenous control
genes

## Slide 4
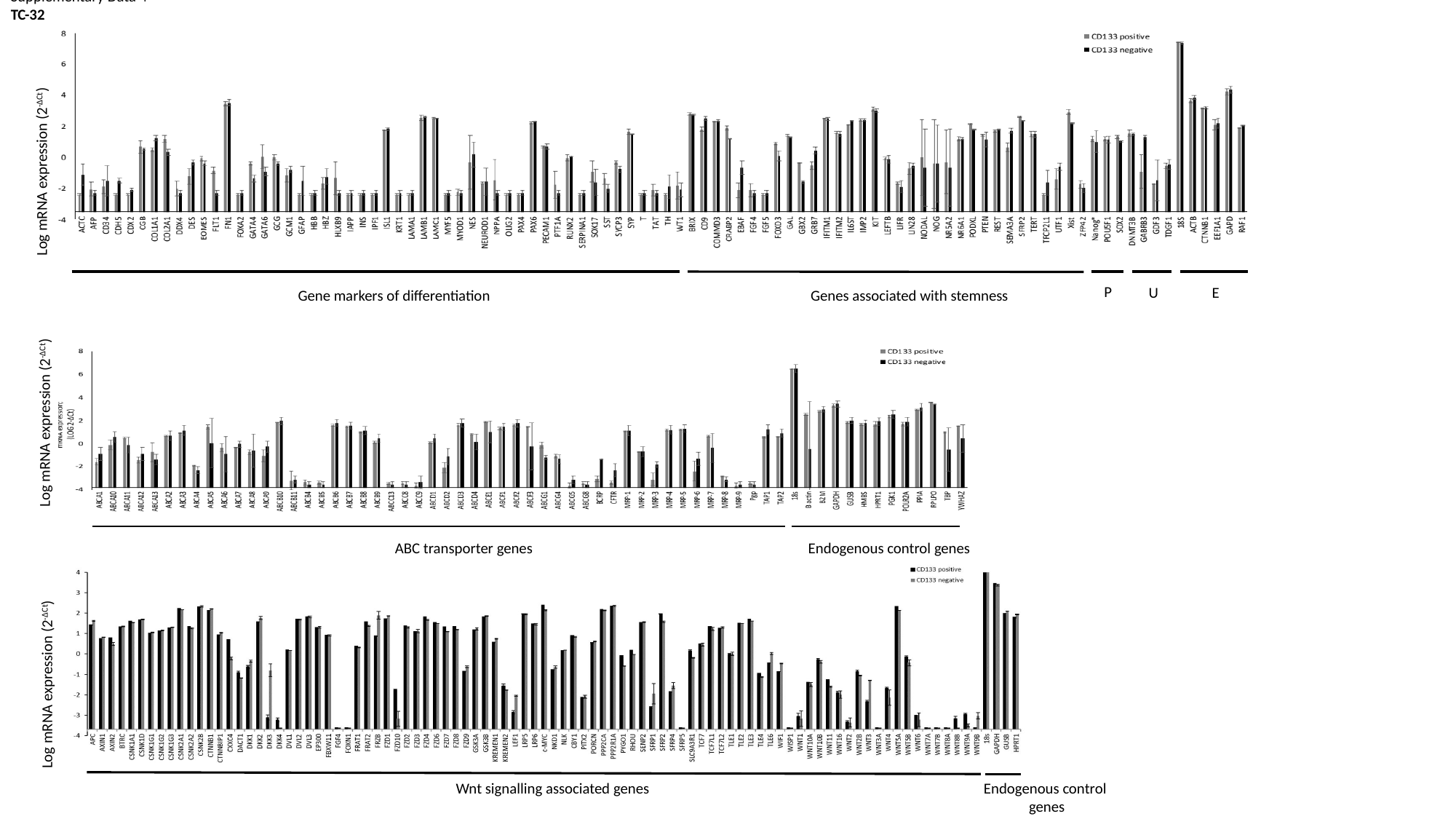

Supplementary Data 4
TC-32
P
U
E
Genes associated with stemness
Gene markers of differentiation
Log mRNA expression (2-∆Ct)
Log mRNA expression (2-∆Ct)
ABC transporter genes
Endogenous control genes
Log mRNA expression (2-∆Ct)
Wnt signalling associated genes
Endogenous control
genes

## Slide 5
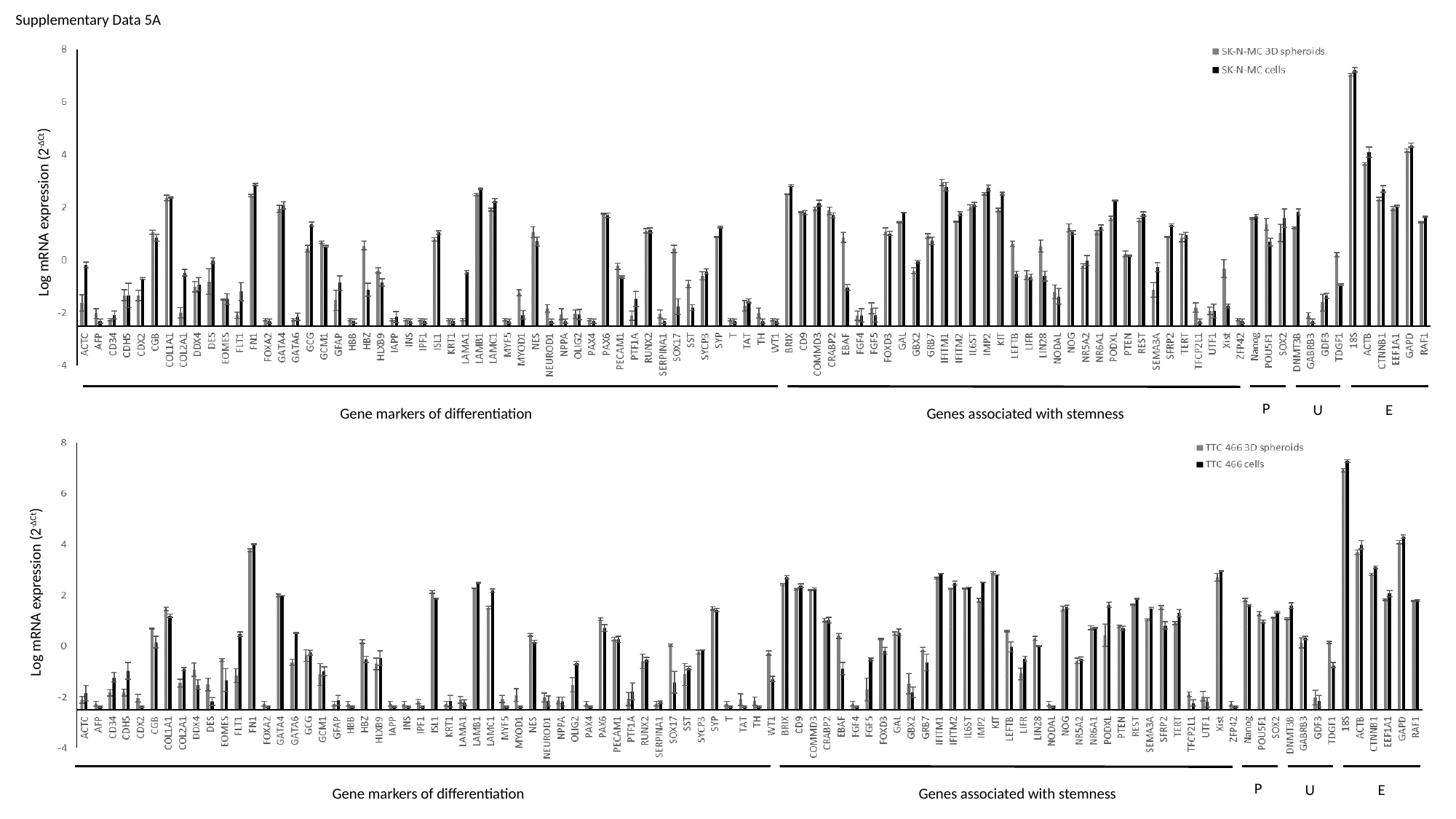

Supplementary Data 5A
Log mRNA expression (2-∆Ct)
P
U
E
Gene markers of differentiation
Genes associated with stemness
Log mRNA expression (2-∆Ct)
P
U
E
Gene markers of differentiation
Genes associated with stemness

## Slide 6
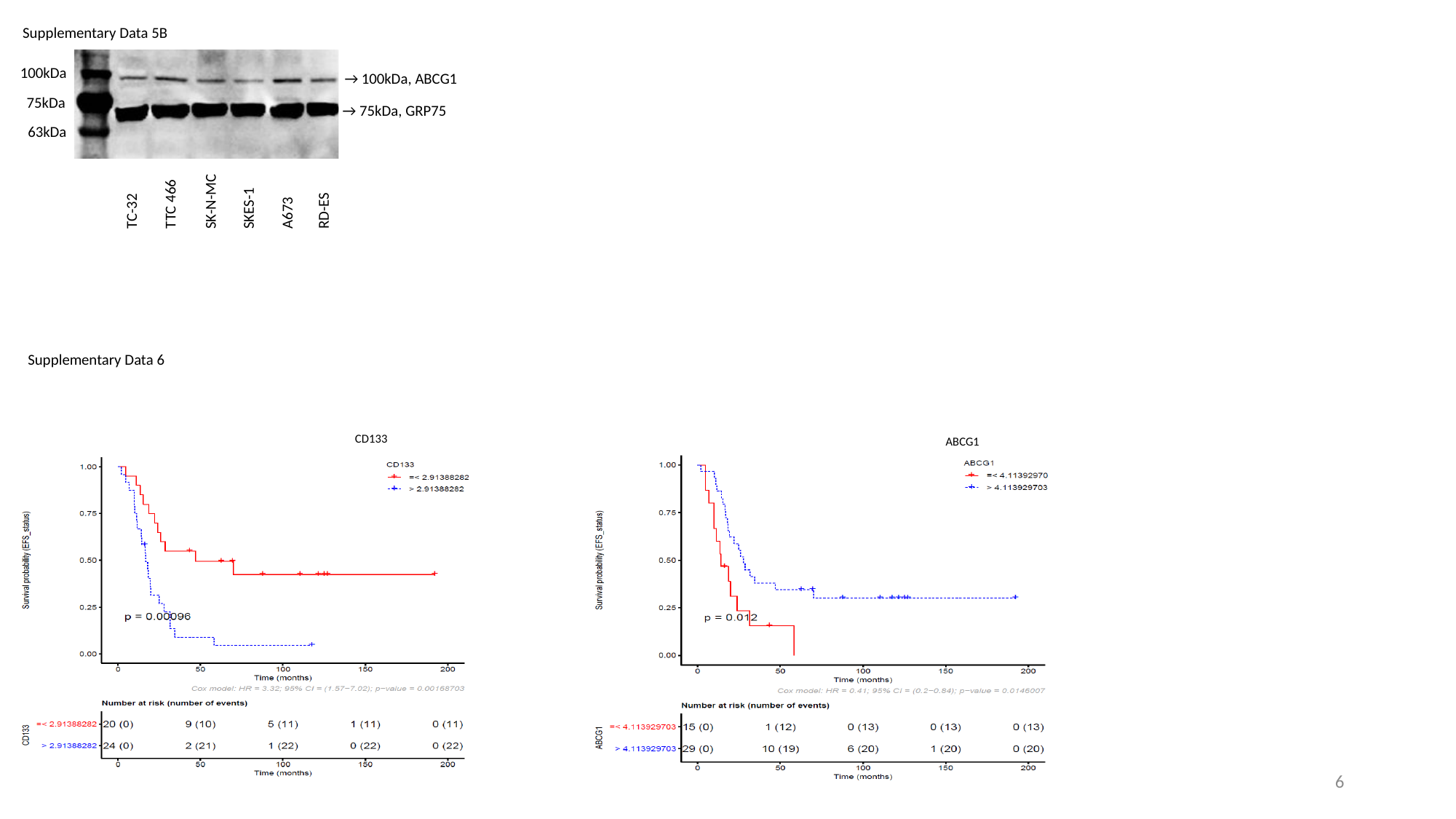

Supplementary Data 5B
100kDa
75kDa
63kDa
TC-32
TTC 466
SK-N-MC
SKES-1
A673
RD-ES
→ 100kDa, ABCG1
→ 75kDa, GRP75
Supplementary Data 6
CD133
ABCG1
6

## Slide 7
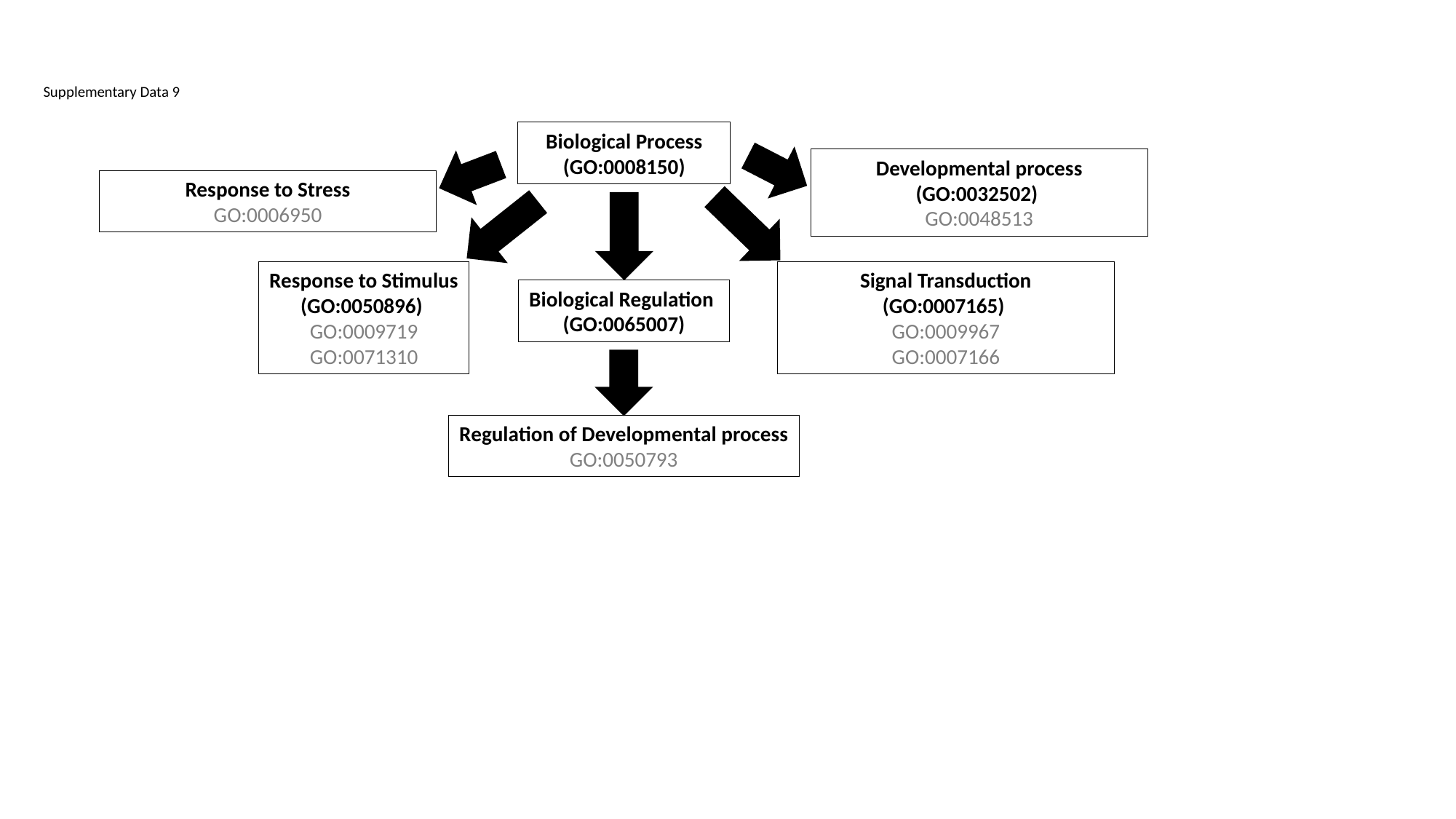

Supplementary Data 9
Biological Process
(GO:0008150)
Response to Stimulus
(GO:0050896)
GO:0009719
GO:0071310
Signal Transduction
(GO:0007165)
GO:0009967
GO:0007166
Biological Regulation
(GO:0065007)
Developmental process
(GO:0032502)
GO:0048513
Response to Stress
GO:0006950
Regulation of Developmental process
GO:0050793
