# Supplementary material for: Exploiting the Stemness and Chemoresistance Transcriptome of Ewing Sarcoma to Identify Candidate Therapeutic Targets and Drug-Repurposing Candidates
Source: Cancers (Basel). 2023 Jan 26;15(3):769. doi: 10.3390/cancers15030769 (PMC9913297; doi:10.3390/cancers15030769)
Supplement: Supplementary file 1 [file cancers-15-00769-s001.zip › Supplementary Methods.pdf]

Supplementary methods

Cellular proliferation

Cells were labelled with the CellTrace™ CFSE Cell Proliferation Kit (Thermo Fisher Scientific) and cellular fluorescence of 10 000 cells analysed at 24h, 48h and 72h on the FACSCalibur [1].

Cell cycle

Cells ($5x10^5$) were fixed in ethanol overnight at -20°C and labelled with propidium iodide (40µg/ml Sigma-Aldrich), containing 3.8mM sodium citrate (VWR International, Leicestershire, UK), 1.5U of RNase Cocktail Enzyme Mix (Applied Biosystems, Thermo Fisher Scientific), in PBS for 3h at 4°C [1]. Labelled cells (100 000) were analysed on the FACSCalibur.

Telomere repeats

DNA was extracted using the DNeasy Blood & Tissue Kit (Qiagen, UK). The quantity and quality of extracted DNA was assessed using the[2]) and the size and fragmentation by electrophoresis (Agilent Technologies 2100 bioanalyzer). The relative telomere length measured by quantitative PCR (qPCR) for telomere repeats relative to a single-copy gene, 36B4 [3]. The difference between the Ct values of the telomere reaction (T) and single copy gene (S) (T/S ratio) was used to generate the average telomere length per genome.

Migration

Migration was determined up to 72h as previously described [1]. Migration Index (MI) = total migrated area relative to the size of the spheroid core at 0h [1].

MRP1 activity

MRP1 dependent efflux activity was measured using the calcein-F efflux assay [4].

Effect of doxorubicin

Cells were treated with increasing concentrations of the MRP1 substrate doxorubicin (3.5-224nM) for 48h and viable cell number counted using the trypan blue exclusion assay (Vi-cell, Beckman Coulter) [5].

References

1. Roundhill, E.A.; Jabri, S.; Burchill, S.A. ABCG1 and Pgp identify drug resistant, self-renewing osteosarcoma cells. *Cancer Lett* **2019**, *453*, 142-157, doi:10.1016/j.canlet.2019.03.011.
2. Brownhill, S.C.; Taylor, C.; Burchill, S.A. Chromosome 9p21 gene copy number and prognostic significance of p16 in ESFT. *Br J Cancer* **2007**, *96*, 1914-1923, doi:6603819 [pii]10.1038/sj.bjc.6603819.
3. O'Callaghan, N.; Dhillon, V.; Thomas, P.; Fenech, M. A quantitative real-time PCR method for absolute telomere length. *Biotechniques* **2008**, *44*, 807-809, doi:10.2144/000112761.

4. Roundhill, E.A.; Burchill, S.A. Detection and characterisation of multi-drug resistance protein 1 (MRP-1) in human mitochondria. *Br J Cancer* **2012**, *106*, 1224-1233, doi:10.1038/bjc.2012.40.
5. Myatt, S.S.; Redfern, C.P.; Burchill, S.A. p38MAPK-Dependent sensitivity of Ewing's sarcoma family of tumors to fenretinide-induced cell death. *Clin Cancer Res* **2005**, *11*, 3136-3148, doi:11/8/3136 [pii]10.1158/1078-0432.CCR-04-2050.
